# Effect of a care transition intervention by pharmacists: an RCT

**DOI:** 10.1186/1472-6963-14-406

**Published:** 2014-09-18

**Authors:** Karen B Farris, Barry L Carter, Yinghui Xu, Jeffrey D Dawson, Constance Shelsky, David B Weetman, Peter J Kaboli, Paul A James, Alan J Christensen, John M Brooks

**Affiliations:** College of Pharmacy, University of Michigan, 428 Church St, Ann Arbor, MI 48109-1065 USA; Department of Pharmacy Practice and Science, College of Pharmacy, University of Iowa, Iowa City, IA USA; Department of Family Medicine, Carver College of Medicine, University of Iowa, Iowa City, IA USA; Department of Biostatistics, College of Public Health, University of Iowa, Iowa City, IA USA; Department of Pharmaceutical Care, University of Iowa Hospital and Clinics, Iowa City, IA USA; Department of Internal Medicine, Carver College of Medicine, University of Iowa, Iowa City, IA USA; Iowa City Veterans Affairs Health Care System, Iowa City, IA USA; Department of Psychology, University of Iowa, Iowa City, IA USA; College of Pharmacy, University of South Carolina, Columbia, SC USA

**Keywords:** Clinical pharmacist, Care transitions, Continuity of care, Medication reconciliation, Medication care plan

## Abstract

**Background:**

Pharmacists may improve medication-related outcomes during transitions of care. The aim of the Iowa Continuity of Care Study was to determine if a pharmacist case manager (PCM) providing a faxed discharge medication care plan from a tertiary care institution to primary care could improve medication appropriateness and reduce adverse events, rehospitalization and emergency department visits.

**Methods:**

*Design*. Randomized, controlled trial of 945 participants assigned to enhanced, minimal and usual care groups conducted 2007 to 2012. *Subjects*. Participants with cardiovascular-related conditions and/or asthma or chronic obstructive pulmonary disease were recruited from the University of Iowa Hospital and Clinics following admission to general medicine, family medicine, cardiology or orthopedics. *Intervention*. The minimal group received admission history, medication reconciliation, patient education, discharge medication list and medication recommendations to inpatient team. The enhanced group also received a faxed medication care plan to their community physician and pharmacy and telephone call 3–5 days post-discharge. Participants were followed for 90 days post-discharge. *Main Outcomes and Measures*. Medication appropriateness index (MAI), adverse events, adverse drug events and post-discharge healthcare utilization were compared by study group using linear and logistic regression, as models accommodating random effects due to pharmacists indicated little clustering.

**Results:**

Study groups were similar at baseline and the intervention fidelity was high. There were no statistically significant differences by study group in medication appropriateness, adverse events or adverse drug events at discharge, 30-day and 90-day post-discharge. The average MAI per medication as 0.53 at discharge and increased to 0.75 at 90 days, and this was true across all study groups. Post-discharge, about 16% of all participants experienced an adverse event, and this did not differ by study group (p > 0.05). Almost one-third of all participants had any type of healthcare utilization within 30 days post-discharge, where 15% of all participants had a 30-day readmission. Healthcare utilization post-discharge was not statistically significant different at 30 or 90 days by study group.

**Conclusion:**

The pharmacist case manager did not affect medication use outcomes post-discharge perhaps because quality of care measures were high in all study groups.

**Trial registration:**

Clinicaltrials.gov registration: NCT00513903, August 7, 2007.

## Background

Transitions across care settings are a critical time to manage medications [1–6]. Up to two-thirds of individuals have unintended medication discrepancies at admission, and medication changes made during the hospitalization are not always conveyed to primary care providers following discharge. Rehospitalization is particularly important today as Medicare policy now reduces payments to health systems for specified conditions where rates do not meet target goals. Thus, improving care during transitions is critical. With medications as one important aspect of care coordination, involving pharmacists in these processes may be helpful [7–10].

Medication reconciliation has been advocated to reduce medication discrepancies [6–8, 11], and is defined as the development of a medication list that is as accurate as possible, which is compared at admission, transfer or discharge, to help ensure correct medications at transitions [11]. However, up-to-date discharge medication lists do not ensure the correct medications are obtained and used. In fact, two recent reviews examined the impact of medication reconciliation [7, 8] and stated that it alone was not likely to improve post-discharge utilization, yet it reduced medication discrepancies, potential adverse drug events and adverse drug events [10, 11]. Hesselink et al. noted that about half the studies showed improvements in adverse events or healthcare utilization post-discharge and noted the difficulty in comparing interventions across studies because of their complexity, lack of detail, varying outcomes and variability in study execution [9]. Thus, numerous well-designed studies have been reported, but the evidence is not consistent, and further studies are needed to examine how discharge processes can be improved to achieve optimal outcomes.

We previously reported the methods for the Iowa Continuity of Care Study [12]. The aim of the Iowa Continuity of Care Study was to determine if a pharmacist case manager (PCM) providing a faxed discharge medication care plan from a tertiary care institution to primary care could improve medication appropriateness and reduce adverse events, rehospitalization and emergency department visits. The major goal was to improve communication links between the tertiary hospital, the primary care physician and community pharmacists, and these providers typically received communication from the institution by mail (primary care physician) or not at all (community pharmacists). The hypotheses were: (1) Medication appropriateness will be improved in patients receiving care from PCM versus usual care; (2) Adverse drug events (ADE) will be lower post-discharge in patients receiving care from PCM versus usual care and (3) Number of readmission, emergency department visits or unscheduled office visits will be lower in patients receiving care from PCM versus usual care.

## Methods

### Design

**Figure 1:**
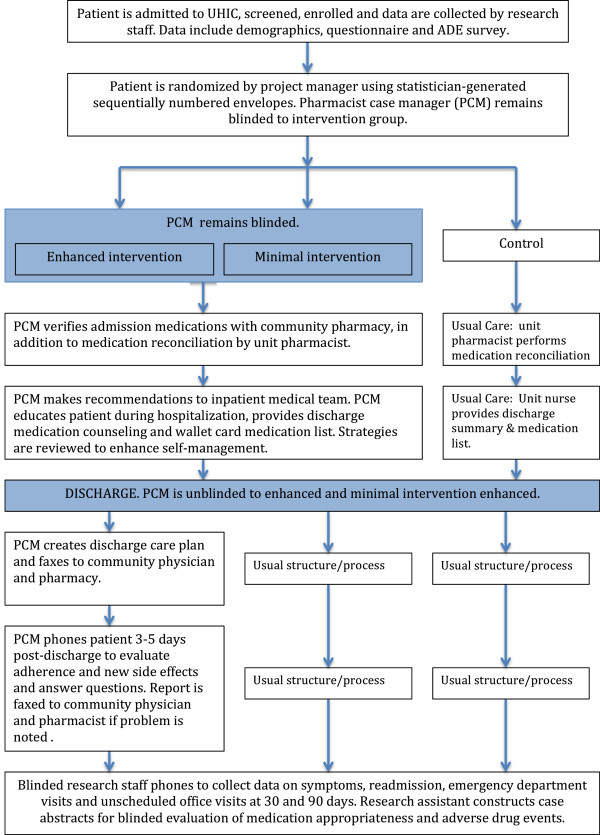
**Iowa Continuity of Care Recruitment and Intervention.**

This study was a randomized, controlled trial where participants were assigned to one of three study groups including enhanced, minimal and control/usual care conducted from 2007 to 2012 (Figure 1) [12]. This study was conducted in one Midwestern academic health center, and the study was approved by University of Iowa and University of Michigan Institutional Review Boards.

### Subjects

Electronic medical records were reviewed, and individuals meeting the inclusion criteria were visited in hospital by trained research assistants to determine eligibility and obtain informed consent. Recruitment occurred Monday-Friday on four services from 2008 through 2011 and we talked with 10–12 admitted patients per week who met the inclusion criteria. Participants were typically enrolled into the study within 1 day after admission and randomized to study group using the statistician-generated blinded randomization scheme with sequentially numbered envelopes. The intervention in the enhanced or minimal intervention groups began immediately after randomization with a visit from a study pharmacist and, if subjects were in the enhanced intervention group, it continued for 3–5 days post-discharge when participants were telephoned. Pharmacists were unaware of whether participants were assigned to the enhanced or minimal intervention group until discharge. For the outcome data, all study participants were followed for 90 days post-discharge.

**Figure 2:**
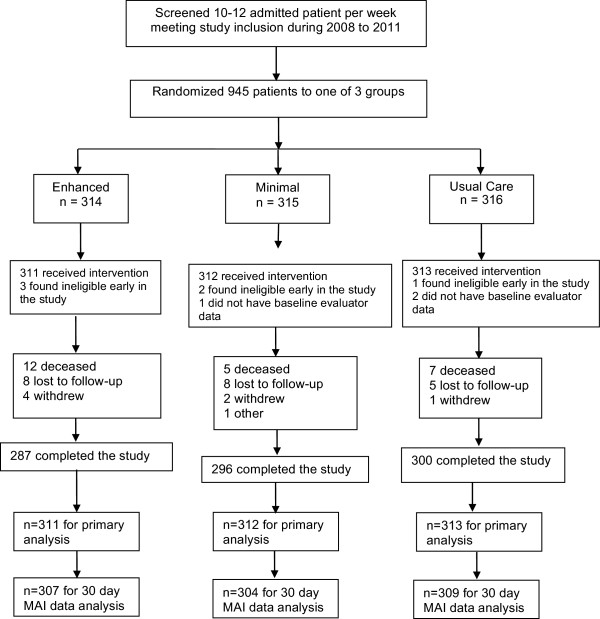
**Iowa Continuity of Care Study Recruitment Chart.**

Participants were recruited from general medicine, family medicine, cardiology or orthopedics. The inclusion criteria were English or Spanish speaker, 18 years or older, admitted with diagnosis of hypertension, hyperlipidemia, heart failure, coronary artery disease, myocardial infarction, stroke, transient ischemic attack, asthma, chronic obstructive pulmonary disease or receiving oral anticoagulation. These conditions were focused on in this study because of previous work we had completed among patients with cardiovascular conditions where pharmacists had impacted their clinical outcomes. Individuals were excluded if they were admitted to psychiatry, surgery or hematology/oncology service, could not use a telephone, had life expectancy <6 months, had dementia or cognitive impairment or had a severe psychiatric diagnosis. These individuals were excluded because they may have had difficulty completing all aspects of the study. In addition, participants were excluded if they received primary care or prescriptions from University of Iowa Hospital and Clinics (UIHC) with shared medical record access. The reason for this exclusion was to include providers external to UIHC to determine if the intervention could improve communication to primary care providers. We randomized 945 participants, and 9 participants (3 from each study group) were either ineligible or lost to follow-up (Figure 2). We stopped the trial at 945, as we had attained 95% of the intended participants and funding was nearing completion. The study was designed to have 80% power to detect a between-group effect size of 0.22 standard deviation for medication appropriateness.

### Intervention

A pharmacist case manager (PCM) provided a set of specified activities for the minimal and enhanced intervention groups, as outlined in Figure 1[12]. The PCMs were PharmD-trained with pharmacy residency training or equivalent direct patient care experience. Two investigators (BLC, AJC) trained two PCMs concerning the intervention, strategies to communicate with inpatient physicians, primary care physicians and community pharmacists and methods to improve medication adherence. Over time there was turnover in PCMs, so each new PCM was trained by the previous intervention pharmacists by providing care together for several study participants, with a focus on the intervention activities and study documentation.

Participants in the minimal and enhanced intervention groups received admission medication reconciliation, pharmacist visits every 2–3 days for patient education during inpatient stay, discharge counseling and discharge medication list. For example, counseling was tailored for each participant and focused on goals of therapy, medication administration, barriers to adherence including cost and patient concerns [12]. Participants in the enhanced intervention group also received a telephone call at 3–5 days post-discharge and primary care physician and community pharmacist received a discharge care plan focused on medication changes and recommendations. The care plan was faxed to the primary care physician and community pharmacist within 24 hours of discharge but usually within 6 hours. The care plan included the discharge medication list, plans for dosage adjustments and monitoring, recommendations for preventing adverse drug events, with patient-specific concerns such as adherence or cost issues highlighted. Usual care was medication reconciliation at admission according to hospital policy, nurse discharge counseling and a discharge medication list for patients. The usual care discharge summary was transcribed and received in the mail by the primary care physician several days or weeks after discharge.

### Data collection

The research assistant gathered baseline data including demographics, clinical characteristics and medication-related information [13–17]. The PCM recorded all interventions. At 30 and 90 days post-discharge, all participants were phoned by trained research assistants to gather self-reported adverse events and symptoms and self-reported healthcare utilization. Primary care provider and pharmacy records were obtained for all subjects. Hospitalization records were obtained from the university hospital and community hospitals when such an event occurred.

The primary outcome for the study was medication appropriateness. The medication appropriateness index (MAI) was completed by two trained research pharmacists (not the intervention pharmacists) who rated the appropriateness of all medications for all study subjects using medication lists at discharge, 30 days post-discharge and 90 days post-discharge [17]. These lists were obtained from community pharmacy and physician office records. Each medication was scored 0 for appropriate or somewhat appropriate, and 1 for inappropriate on each of six criteria. These six criteria (weight) included indication for medication (3), optimal medication (3), dose appropriate (2), drug-disease interaction (2), unnecessary duplication (1) and least expensive alternative (1). For each medication, the weighted sum across the six criteria were calculated, with an MAI range for each medication of 0 = fully appropriate to 12 = fully inappropriate. We calculated the sum per patient and the average MAI per medication.

Secondary outcomes included adverse events (AEs), preventable adverse events and a composite variable of combined hospital readmission, emergency department visit or unscheduled office visit during the 30-day and 90-day follow-up period. We determined adverse events arising from lack of medications, either non-adherence or under-treatment, and adverse drug events arising from medication exposures [18, 19]. The trained research pharmacists reviewed all records including hospital discharge summary, primary care records, pharmacy records and 30-day and 90-day telephone calls to identify symptomatology which we termed potential adverse events. The research pharmacists then rated four questions for each potential adverse event to determine the confidence that the symptoms arose from patient non-adherence, under-treatment or medication exposure and the final question asked about preventability. For medication exposure, a Naranjo score was calculated from ten items to determine the extent to which symptoms may be related to medications [20]. The research pharmacists’ ratings to these questions were reviewed by a team of physician and pharmacist to confirm the ratings. In the analysis, only AEs or ADEs with virtual certainty or strong level of confidence or a Naranjo score of 5 or greater was counted. The number of AEs and ADEs for each subject was determined, and each AE and ADE was labeled as preventable if the preventability rating was definite or probable.

Finally, we counted the number of occurrences of hospital readmission, emergency department visits and unscheduled office visits using patient self-report and physician and hospital records.

### Analysis

Descriptive statistics were calculated for the demographics, clinical characteristics and the study outcomes. The fidelity of pharmacist interventions was compared between minimal and enhanced groups using the chi-square test for categorical variables and t-tests for numeric variables. Preliminary analyses accommodating random effects due to pharmacists indicated very little, if any, clustering within pharmacist, so standard regression methods were used to analyze the study outcomes. For the case of MAI and MAI per medication, linear regression was performed on the square root scale to improve normality. The other outcomes were dichotomous and analyzed using logistic regression. Since the minimal and enhanced interventions were identical until patient discharge, comparisons were made with both intervention groups combined versus the control group at discharge. For post-discharge comparisons, each intervention group was compared to the control group. We performed a subgroup analysis including only those who had heart failure or asthma/pulmonary disease using the same analytic approach as described above.

## Results

We enrolled 945 participants into the study (Figure 2), and study groups were comparable at baseline (Table 1). The mean age (±SD) of participants in the study was 61.0 (±12.2) years, with 91% white and 66% married or living as married. The income and education distributions showed that 47% of study participants had an annual income less than $40,000 and 49% had a high school education, respectively. Most participants in the study had health insurance, with almost half having private insurance. Almost all, 96%, had prescription drug insurance.

**Table 1:** **Participant demographic and clinical characteristics**

	Enhanced	Minimal	Control	Total
	N = 311	N = 312	N = 313	(n = 936)
**Age**				
≤44 years	34 (10.9)	38 (12.2)	29 (9.3)	101 (10.8)
45–54 years	43 (13.8)	52 (16.7)	49 (15.7)	144 (15.4)
55–64 years	101 (32.5)	96 (30.8)	110 (35.1)	307 (32.8)
65–74 years	85 (27.3)	85 (27.2)	85 (27.2)	255 (27.2)
≥75 years	48 (15.4)	41 (13.1)	40 (12.8)	129 (13.8)
**Education**				
Less than high school (1–8)	16 (5.1)	12 (3.9)	22 (7.0)	50 (5.3)
High school (9–12)	151 (48.6)	153 (49.0)	151 (48.2)	455 (48.6)
Some college	66 (21.2)	73 (23.4)	59 (18.9)	198 (21.2)
College degree	45 (14.5)	41 (13.1)	54 (17.3)	140 (15.0)
Professional or advanced degree	33 (10.6)	33 (10.6)	27 (8.6)	93 (9.9)
**Race**				
White	287 (92.3)	283 (90.7)	285 (91.1)	855 (91.4)
African American	12 (3.9)	16 (5.1)	18 (5.8)	46 (4.9)
Hispanic	7 (2.3)	8 (2.6)	6 (1.9)	21 (2.2)
Other	5 (1.6)	5 (1.6)	4 (1.3)	14 (1.5)
**Annual income**				
<$10,000	32 (10.4)	34 (11.0)	35 (11.2)	101 (10.9)
$10,000 - $24,999	55 (17.8)	56 (18.1)	64 (20.5)	175 (18.8)
$25,000 - $39,999	68 (22.0)	53 (17.1)	42 (13.5)	163 (17.5)
$40,000 - $54,999	56 (18.1)	46 (14.8)	42 (13.5)	144 (15.5)
$55,000 and greater	98 (31.7)	121 (39.0)	129 (41.4)	348 (37.4)
Missing	2	2	1	5
**Marital status**				
Single/never married	21 (6.8)	18 (5.8)	29 (9.3)	68 (7.3)
Married or living as married	194 (62.6)	213 (68.3)	206 (65.8)	613 (65.6)
Divorced/separated	56 (18.1)	49 (15.7)	51 (16.3)	156 (16.7)
Widowed	39 (12.6)	32 (10.3)	27 (8.6)	98 (10.5)
**Medical service**				
Internal Medicine/Family Medicine	79 (25.4)	84 (26.9)	84 (26.8)	247 (26.4)
Cardiology	111 (35.7)	111 (35.6)	112 (35.8)	334 (35.7)
Orthopedics	121 (38.9)	117 (37.5)	117 (37.4)	355 (37.9)
**Type of medical insurance**				
Private	157 (50.5)	145 (46.5)	158 (50.5)	460 (49.2)
Medicare	120 (38.6)	123 (39.2)	114 (36.4)	357 (38.1)
Medicaid	27 (8.7)	39 (12.5)	38 (12.1)	104 (11.1)
Other insurer/none/self-pay	7 (2.3)	5 (1.6)	3 (1.0)	15 (1.6)
**Prescription drug insurance**				
Yes	297 (95.5)	294 (94.5)	303 (97.1)	894 (95.7)
Average number of prescription medications*	11.0 (5.7)	11.8 (6.0)	10.4 (5.5)	11.0 (5.8)
**Chronic conditions (% yes)**				
Hypertension	249 (80.1)	231 (74.0)	239 (76.4)	719 (76.8)
Hyperlipidemia	194 (62.4)	195 (62.5)	189 (60.3)	578 (61.8)
Heart failure	85 (27.3)	91 (29.2)	76 (24.3)	252 (26.9)
Coronary artery disease	114 (36.7)	108 (34.6)	94 (30.0)	316 (33.8)
Myocardial infarction	69 (22.2)	68 (21.8)	62 (19.8)	199 (21.2)
Transient ischemic attacks	26 (8.4)	30 (9.6)	26 (8.3)	82 (8.8)
Stroke	12 (3.9)	17 (5.5)	14 (4.5)	43 (4.6)
Depression	105 (33.8)	105 (33.7)	98 (31.3)	308 (32.9)
Anxiety	91 (29.3)	79 (25.3)	73 (23.3)	243 (26.0)
Arthritis	193 (62.1)	197 (63.1)	192 (61.3)	582 (62.2)
Diabetes	113 (36.3)	123 (39.4)	114 (36.4)	350 (37.4)
Kidney disease*	35 (11.3)	58 (18.6)	34 (10.9)	127 (13.6)
Liver disease	15 (4.8)	17 (5.5)	14 (4.5)	46 (4.9)
Asthma or pulmonary disease	79 (25.4)	90 (28.9)	86 (27.5)	255 (27.2)
Knee replacement	82 (26.4)	70 (22.4)	78 (24.9)	230 (24.6)
Hip replacement	49 (15.8)	36 (11.5)	32 (10.2)	117 (12.5)
Fracture	135 (43.4)	133 (42.6)	131 (41.9)	399 (42.6)
Cancer	56 (18.0)	54 (17.3)	49 (15.7)	159 (17.0)
Other	172 (55.3)	183 (58.7)	171 (54.6)	526 (56.2)
**Smoking status**				
Never	132 (42.4)	150 (48.1)	126 (40.3)	408 (43.6)
Current	33 (10.6)	35 (11.2)	27 (8.6)	95 (10.2)
Ex-smoker	146 (47.0)	127 (40.7)	160 (51.1)	433 (46.3)
**Alcohol intake**				
None	200 (64.3)	190 (60.9)	183 (58.7)	573 (61.3)
<1 drink/day	82 (26.4)	95 (30.5)	100 (32.1)	277 (29.6)
1–2 drinks/day	22 (7.1)	21 (6.7)	24 (7.7)	67 (7.2)
≥3 drinks/day	7 (2.3)	6 (1.9)	5 (1.6)	18 (1.9)
**Self-rated health**				
Excellent	15 (4.8)	13 (4.2)	19 (6.1)	47 (5.0)
Very good	55 (17.7)	44 (14.1)	48 (15.3)	147 (15.7)
Good	116 (37.4)	122 (39.1)	132 (42.2)	370 (39.6)
Fair	88 (28.4)	87 (27.9)	79 (25.2)	254 (27.2)
Poor	36 (11.6)	46 (14.7)	35 (11.2)	117 (12.5)
**Self-reported medication adherence**				
Forget (% never or rarely)*	233 (75.2)	245 (79.0)	267 (85.6)	745 (79.9)
Careless (% never or rarely)	282 (91.0)	289 (93.2)	299 (95.8)	870 (93.4)
Stop if feel better (% never or rarely)	292 (94.2)	292 (94.2)	302 (96.8)	886 (95.1)
Stop if feel worse (% never or rarely)	278 (89.7)	278 (89.7)	286 (91.7)	842 (90.3)
**Medication management ability**				
Able (% scoring 5)	203 (66.3)	218 (70.1)	211 (68.5)	632 (68.3)
Some limitation (% 4 or less)	103 (33.7)	93 (29.9)	97 (31.5)	293 (68.3)
Missing	5	1	5	11
**Medication self-efficacy scale**				
Average (standard deviation)	123.3 (±12.5)	124.4 (±12.3)	125.0 (±10.1)	124.2 (±11.7)
**Instrumental activities of daily living**				
No limitation	215 (69.4)	226 (72.4)	239 (76.4)	680 (72.7)
Requires help or unable on 1	35 (11.3)	42 (13.5)	27 (8.6)	104 (6.0)
Requires help or unable on 2	22 (7.1)	13 (4.2)	21 (6.7)	56 (6.0)
Requires help or unable on 3 or more	38 (12.2)	31 (9.9)	26 (8.3)	95 (10.1)

*Variables were significantly different among groups (p < 0.01).

Participants were similar in terms of chronic conditions, smoking status and alcohol intake at baseline, though baseline medications were slightly higher in the minimal intervention group compared to controls (p = 0.0009) (Table 1). The prevalence of chronic kidney disease and reporting never or rarely forgetting to take medications did vary by study group. More participants in the control group (85.6%) rarely forgot their medications compared to minimal (79.0%) and enhanced (75.2%) (p < 0.0046). Self-rated health was comparable across the study groups, and 13% rated their health as poor, 27% as fair, 40% as good and 21% were very good or excellent.

Intervention fidelity was high for admission medication reconciliation and wallet card, but was variable for other parts of the intervention (Table 2). Discharge counseling was provided to 75% of enhanced and minimal intervention participants. Among the enhanced intervention group, 84% had their care plan faxed to their community physicians and 80% had it faxed to community pharmacists. Five pharmacists delivered the PCM activities over the study period, and their average time spent on study activities varied from 83 (±26) to 202 (±112) minutes per patient per pharmacist (p < 0.0001). As expected, pharmacists spent considerably more time with enhanced versus minimal participants (p < 0.0001).

**Table 2:** **Fidelity of pharmacists’ interventions**

	Enhanced	Minimal	P-values
	n = 311	n = 312	
Admission medication reconciliation	311 (100%)	312 (100%)	
Community pharmacy contacted	300 (96.5%)	305(97.8%)	0.34
Discharge counseling completed	235 (75.6%)	235 (75.3%)	0.94
Wallet card completed	309 (99.4%)	308 (98.7%)	0.41
Medication issues identified in hospital	275 (88.4%)	249 (79.8%)	0.003
Post-discharge phone call completed	301 (96.8%)	4 (1.3%)*	
Discharge care plan faxed to community physician	267 (85.9%)	1 (0.3%)*	
Discharge care plan faxed to community pharmacist	246 (79.1%)	1 (0.3%)*	
Discharge care plan included medication recommendations to community physician	207 (66.6%)	NA	
Discharge care plan medication issues identified by pharmacists†	To Hospital & Community Physicians	To Hospital Physicians	
Mean (±SD)	6.6 (±6.8)	3.2 (±4.0)	
Total number of issues identified	2063	1012	
Dosing or administration	260	131	
Indication	754	363	
Efficacy	319	101	
Cost	103	38	
Risk to patient	627	379	
Discharge care plan recommendations made to physicians†	To Hospital & Community Physicians	To Hospital Physicians	
Mean (±SD)	7.1 (±6.6)	3.5 (±3.8)	
Total number of recommendations	2220	1077	
Discontinue medications	377	195	
Add medications	566	256	
Change medications	361	151	
Disease monitoring	280	56	
Follow-up patient	262	134	
Patient education	283	239	
Adherence education	91	46	
Time pharmacist spent on each patient (minutes)	210.0 (±93.0)	118.5 (±58.6)	<.0001

*Inadvertent crossover since care plans should not have been sent according to randomization.

†Many but not all medication issues and recommendations were repeated to the community physicians, accounting for almost twice the numbers in the enhanced group.

The average MAI per medication as 0.53 at discharge and increased to 0.75 at 90 days, and this was true across all study groups (Table 3). There were no statistically significant differences in MAI supporting the intervention. The control group had a statistically significantly lower (improved) total MAI at discharge compared to minimal and enhanced groups, but the average MAI per medication was not different.

**Table 3:** **Medication Appropriateness Index (MAI) by study group**

	Enhanced	Minimal	Control	P-values ^*^
**Summed MAI per Participant**				
Discharge	7.1 (±7.0)	8.0 (±8.4)	6.1 (±6.6)	E + M vs. C: 0.03
30 days post-discharge	10.1 (±8.9)	11.7 (±11.2)	9.6 (±9.5)	E vs. C: 0.78
				M vs. C: 0.07
90 days post-discharge	11.6 (±10.5)	13.6 (±12.3)	11.1 (±11.3)	E vs. C: 0.94
				M vs. C: 0.02
**MAI per Medication**				
Discharge	0.52 (±0.53)	0.55 (±0.57)	0.51 (±0.54)	E + M vs. C: 0.26
30 days post-discharge	0.62 (±0.50)	0.69 (±0.61)	0.65 (±0.57)	E vs. C: 0.86
				M vs. C: 0.70
90 days post-discharge	0.72 (±0.68)	0.80 (±0.65)	0.73 (±0.63)	E vs. C: 0.84
				M vs. C: 0.33

*E = enhanced, M = minimal and C = control groups.

Post-discharge, about 16% of all participants experienced an AE, and this did not differ by study group (p > 0.05) (Table 4). The enhanced (6.1%) and minimal (6.1%) groups had more AE and ADEs during hospitalization identified than controls (4.5%), but the difference was not statistically significant.

**Table 4:** **Adverse Events (AE) from non-adherence and under-treatment and Adverse Drug Events (ADE) by study group**

	Enhanced	Minimal	Control	P-values ^*^
**Initial Hospitalization**	n = 311	n = 312	n = 313	
Number of possible adverse events	40	43	40	
Number of AEs due to medication non-adherence†	9	9	6	
Number of AEs due to medication under-treatment†	1	3	3	
Number of ADEs†	15	16	15	
Percent of participants with any AE or ADE†	18 (5.8%)	19 (6.1%)	22 (7.0%)	E + M vs. C: 0.52
**During Hospitalization**				
Number of possible adverse events	55	52	57	
Number of AEs due to medication non-adherence †	1	2	0	
Number of preventable medication non-adherence AEs‡	1	2	0	
Number of AEs due to medication under-treatment †	2	0	1	
Number of preventable medication under-treatment AEs‡	2	0	1	
Number of ADEs†	18	27	16	
Number of preventable ADEs‡	0	3	1	
Percent of participants with any AE or ADE†	19 (6.1%)	19 (6.1%)	14 (4.5%)	E + M vs. C: 0.31
**Post-Discharge**	n = 306	n = 309	n = 311	
Number of possible adverse events	183	180	174	
Number of AEs due to medication non-adherence†	15	8	10	
Number of preventable medication non-adherence AEs‡	15	7	9	
Number of AEs due to medication under-treatment†	10	13	6	
Number of preventable medication under-treatment AEs‡	6	11	5	
Number of ADEs†	47	49	60	
Number of preventable ADEs‡	8	7	9	
Percent of participants with any AE or ADE†	48 (15.7%)	50 (16.2%)	53 (17.0%)	E vs. C: 0.72
				M vs. C: 0.95

*E = enhanced, M = minimal and C = control groups.

†Medication non-adherence and under-treatment were labeled adverse events (AEs) and counted only if the rating was “Virtually certain” or “Strong level of confidence”. ADEs were counted only if the Naranjo score was 5 or greater.

‡AEs and ADEs were considered preventable if the rating was “Definitely preventable” or “Probably preventable”.

About 29% of all participants had any type of healthcare utilization within 30 days post-discharge, where 15% of all participants had a 30-day readmission. There were no statistically significant differences by study group for any utilization outcomes (Table 5). We examined the study participants with CHF and/or asthma/COPD separately, and the results were consistent with our overall findings (data not shown).

**Table 5:** **Hospital readmission, emergency department visits or unscheduled office visits by study group**

	Enhanced	Minimal	Control	*P-values
**Number of patients with any post-discharge healthcare use**				
30 days	81 (28.8%)	88 (29.5%)	87 (29.6%)	E vs. C: 0.82
				M vs C: 0.92
90 days	97 (34.5%)	90 (30.5%)	88 (29.9%)	E vs. C: 0.20
				M vs C: 0.62
**Number of patients with at least one specific post-discharge healthcare use**				
30 day readmission	47(16.7%)	40 (13.4%)	43 (14.6%)	E vs. C: 0.29
				M vs C: 0.38
30 day emergency dept visit	38 (13.5%)	49 (16.5%)	52 (17.8%)	E vs. C: 0.18
				M vs C: 0.71
30 day unscheduled visit	31 (11.0%)	30 (10.1%)	32 (10.9%)	E vs. C: 0.81
				M vs C: 0.69
90 day readmission	49 (17.4%)	51 (17.3%)	47 (16.0%)	E vs. C: 0.77
				M vs C: 0.83
90 day emergency dept visit	41 (14.6%)	40 (13.6%)	46 (15.7%)	E vs. C: 0.99
				M vs C: 0.54
90 day unscheduled visit	42 (15.0%)	36 (12.2%)	33 (11.3%)	E vs. C: 0.18
				M vs C: 0.74

*E = enhanced, M = minimal and C = control groups.

## Discussion

The pharmacist case manager did not affect medication appropriateness, number of clinically-relevant adverse events or adverse drug events or post-discharge healthcare utilization. These findings might be explained by the overall good medication appropriateness and generally low re-hospitalizations, ED visits and unscheduled office visits in all study groups when compared to previous studies. Other studies that have evaluated PCMs had mixed results, but our methodology and measures were anticipated to be sensitive to the intervention given previous findings about pharmacists’ impact post-discharge [10]. Our finding is important given our strong study design, subjects with high medication use and a comprehensive assessment of all study outcomes.

Our primary outcome was medication appropriateness and we had good power to detect meaningful differences based on prior studies (17). Our study was powered to detect effect sizes of 0.22 in the MAI, but the largest effect size seen was 0.21 for minimal vs. control at 90 days, and this was in the wrong direction (minimal having a higher value). Moreover, the MAI per medication ranged from 0.51 to 0.8, indicating high medication appropriateness across the study. These finding suggest very good medication appropriateness with limited opportunity for improvement regardless of the intervention.

Our process to determine adverse events and adverse drug events was comprehensive using patient self-report of symptomatology along with patient medical records and expert opinion to establish attribution [18–20]. Forster and colleagues evaluated ADEs after discharge [21]. Of 400 patients, 45 (11%) had an ADE and over half could have been prevented or ameliorated. These ADEs occurred in spite of electronic discharge summaries being transmitted to primary care physicians. In our study, about 16% of all participants experienced an adverse event within the 90-day post-discharge period that was virtually certain or highly likely attributed to medications. While an interim analysis showed that medication discrepancies were reduced in the enhanced group, and the medication list in the primary care physician office was more likely to be up-to-date compared to the minimal or control groups [22], this finding did not translate into other findings.

Between 2008 and 2010 the readmission at the study hospital were 18.2 and 19.1%, respectively, when our study was conducted [23]. However, the 30-day readmission rate among study participants ranged from 13.4% to 16.7%, which is lower than anticipated when the study was designed. These observations would suggest that the rate of re-hospitalizations had dropped substantially after the study was designed which made it difficult for the intervention to improve these rates. There is conflicting evidence from other studies whether 30-day readmission rates were declining overall during our study period 2007–2012. Findings in the Veterans Affairs institutions suggest a decreasing trend over the past decade, but rates were steady for Medicare fee-for-service beneficiaries from 2007 through 2011, until 2012 when rates declined [23–25]. Focusing this intervention exclusively on individuals with high risk for readmission post-discharge would have been a better strategy, yet our sub-analysis for heart failure showed no differences. Finally, a broader view of health and functional status besides medications is likely necessary to further reduce readmissions. For example, medications are one component of the Care Transitions Intervention, but it is not the solitary focus [26].

The content of the PCM intervention was multi-faceted not merely medication reconciliation. There was some variability in the delivery of the intervention by the PCMs, but almost all participants were provided a medication reconciliation at admission and discharged medication lists. The PCM was expected to improve medication use at discharge via medication reconciliation and recommendations to the inpatient team. During study initiation, policy changed at the study hospital and many usual care patients received admission medication reconciliation. While the PCM contacted the community pharmacist for almost all participants at baseline in the minimal and enhanced groups, we cannot establish the effect of this call. As well, the discharge care plan was faxed to 86% of participants’ community physicians, and only 66% contained specific medication recommendations. These two facets of the intervention may have reduced its effectiveness.

Numerous studies have shown a positive impact of pharmacists’ recommendations when they work directly with teams [27–34]. In considering the lack of effect at discharge, the PCMs were study pharmacists and not part of the inpatient medical teams. Our interim analysis showed that about half of the pharmacists’ recommendations were accepted by hospitalists or physicians in the in-patient setting [35] and the recommendations did not change the prescribing of cardiovascular medications [36]. Anecdotally, inpatient physicians often were reluctant to accept recommendations for chronic conditions, e.g. hyperlipidemia or diabetes, when these were not the reason for the hospitalization. These findings were disappointing, and the low rate of accepted recommendations is counter to pharmacists’ contribution to improved outcomes in both inpatient and primary care settings [27–34].

The medication care plan and the 3–5 day post-discharge telephone call were expected to have an impact post-discharge. About 86% of participants had the discharge care plan faxed to physicians and almost all participants in the enhanced intervention group received the post-discharge telephone follow-up. While over 2000 recommendations were in the care plans, about one-half of these were provided to community physicians. Many of the recommendations were related to adding, changing or discontinuing medications. Physicians in the community may have not been aware of the hospitalization until a follow-up visit was done post-discharge. At that time, a discharge summary and/or the medication care plan could have been reviewed, but we have no conclusive way to know whether that occurred. There was no verbal hand-off back to the community providers. The development of a discharge medication care plan and sharing the care plan with physicians via fax were insufficient to improve medication use or patient outcomes. A verbal hand-off to physicians was effective in two previous studies, suggesting this contact may be critical [37, 38]. However, the best time to actually complete the verbal hand-off to the primary care physician is unclear, as the timing would likely be best when the provider is scheduled to see the discharged patient. Further, the telephone follow-up, although provided to almost all intended participants at 3–5 days post-discharge, was not sufficient to improve outcomes for this population.

Finally, the study population was younger than many other studies where care transition programs have been effective [9, 26]. Many of the individuals may not have had a high enough acuity to require such support during their transition, as about 70% had no instrumental activity of daily living limitation, only 40% rated their health as fair or poor, few reported intentional medication non-adherence, 25% reported forgetting medications and most participants had high medication self-efficacy. In retrospect, we should have focused this care transition intervention towards individuals with transition issues rather than those where pharmacists had been successful impacting outcomes in previous studies. In consideration of other studies and our current findings, we suggest that medication-focused care transition activities be targeted to individuals with greater health acuity who are known to exhibit medication management difficulties. In addition, implementation of medication-related recommendations will require more intense interventions than fax from the tertiary hospital with the primary care physicians after discharge.

This study has implications for policies related to reducing hospital readmissions. Hospitals will need to evaluate the most efficient and effective methods to reduce re-admissions and reduce adverse events. Improved information technology may be useful in transmitting discharge care plan information more efficiently, but it appears likely that a verbal or electronically verified hand-off may be necessary [37, 38]. Such an approach would help ensure that important issues requiring follow-up were specifically identified for community providers. However, the most effective strategy may be stronger relationships and communications with care navigators or primary care physicians to optimize medications since it is hospitals that are at risk for re-admissions.

This study has limitations. At baseline, forgetting medications was not well randomized. Yet, it is unlikely that this single aspect of medication management would change the impact of the intervention on medication appropriateness or adverse events to a great degree across the three study groups. The intervention fidelity was good but not without some issues. We cannot separate the effect of any specific component of the intervention such as patient counseling on the outcomes of the study. We failed to determine whether community physicians actually used the discharge medication care plan information that was sent, and this is an important missing link in the process of care and in this study. As well, factors such as pharmacist personalities and health-system relationships with non-health system primary care providers form an important context in which such studies are completed and these cannot be ignored when considering generalizability. We did not separate self-reported versus medically document post-discharge events, which may contribute to measurement error making it more difficult to detect an effect.

## Conclusions

In conclusion, a pharmacist case manager providing admission medication reconciliation, in-patient medication recommendations to the hospital team, patient education, post-discharge information to community physicians and post-discharge telephone call to patients did not affect medication appropriateness, adverse events or post-discharge healthcare utilization.

## Abbreviations

PCM: Pharmacist case manager
ADE: Adverse drug event
UIHC: University of Iowa Hospitals and Clinics
MAI: Medication appropriateness index
AE: Adverse event
SD: Standard deviation.
